# Understanding Gaps in the Hypertension and Diabetes Care Cascade: Systematic Scoping Review

**DOI:** 10.2196/51802

**Published:** 2024-02-16

**Authors:** Jie Wang, Fangqin Tan, Zhenzhong Wang, Yiwen Yu, Jingsong Yang, Yueqing Wang, Ruitai Shao, Xuejun Yin

**Affiliations:** 1 School of Population Medicine and Public Health Chinese Academy of Medical Sciences & Peking Union Medical College Beijing China; 2 The George Institute for Global Health University of New South Wales Sydney Australia

**Keywords:** care cascade, hypertension, diabetes, scoping review, hypertension and diabetes care, review

## Abstract

**Background:**

Hypertension and diabetes are global health challenges requiring effective management to mitigate their considerable burden. The successful management of hypertension and diabetes requires the completion of a sequence of stages, which are collectively termed the care cascade.

**Objective:**

This scoping review aimed to describe the characteristics of studies on the hypertension and diabetes care cascade and identify potential interventions as well as factors that impact each stage of the care cascade.

**Methods:**

The method of this scoping review has been guided by the framework by Arksey and O’Malley. We systematically searched MEDLINE, Embase, and Web of Science using terms pertinent to hypertension, diabetes, and specific stages of the care cascade. Articles published after 2011 were considered, and we included all studies that described the completion of at least one stage of the care cascade of hypertension and diabetes. Study selection was independently performed by 2 paired authors. Descriptive statistics were used to elucidate key patterns and trends. Inductive content analysis was performed to generate themes regarding the barriers and facilitators for improving the care cascade in hypertension and diabetes management.

**Results:**

A total of 128 studies were included, with 42.2% (54/128) conducted in high-income countries. Of them, 47 (36.7%) focused on hypertension care, 63 (49.2%) focused on diabetes care, and only 18 (14.1%) reported on the care of both diseases. The majority (96/128, 75.0%) were observational in design. Cascade stages documented in the literature were awareness, screening, diagnosis, linkage to care, treatment, adherence to medication, and control. Most studies focused on the stages of treatment and control, while a relative paucity of studies examined the stages before treatment initiation (76/128, 59.4% vs 52/128, 40.6%). There was a wide spectrum of interventions aimed at enhancing the hypertension and diabetes care cascade. The analysis unveiled a multitude of individual-level and system-level factors influencing the successful completion of cascade sequences in both high-income and low- and middle-income settings.

**Conclusions:**

This review offers a comprehensive understanding of hypertension and diabetes management, emphasizing the pivotal factors that impact each stage of care. Future research should focus on upstream cascade stages and context-specific interventions to optimize patient retention and care outcomes.

## Introduction

Noncommunicable diseases (NCDs) constitute a formidable global health challenge, accounting for approximately 80% of NCD-related deaths, and they include cardiovascular diseases, cancers, chronic respiratory diseases, and diabetes [1]. Hypertension is the most pivotal risk factor for cardiovascular diseases [2]. The prevalence of hypertension among adults aged 30-79 years worldwide is estimated to be 1.28 billion, with an alarming 700 million individuals unaware of their condition. Less than half of adults with hypertension are diagnosed and treated. Only approximately 1 in 5 adults with hypertension have their blood pressure controlled [3]. Similarly, the global prevalence of diabetes among adults has surged to 537 million in 2021, with nearly half of these cases (240 million) remaining undiagnosed. Moreover, the treatment rate for diabetes is suboptimal, with only 32.9% of patients receiving appropriate care and a mere 16.5% attaining treatment goals [4]. Evidence suggests that a substantial proportion of patients with hypertension and diabetes reside in low- and middle-income countries (LMICs), wherein the management of these conditions remains persistently low [3,5].

Hypertension and diabetes are often approached differently by distinct clinical subspecialties owing to their clinical complexities. However, it is essential to recognize that the management of these 2 conditions together can be highly beneficial owing to their shared risk factors and bidirectional interaction. The management of hypertension and diabetes also shares the same pathway, which includes early detection, appropriate treatment, and continuous monitoring. The health care systems and implementation strategies designed to ensure the continuity of care exhibit significant overlap and can be harnessed efficiently and effectively to support both hypertension and diabetes patients. The care cascade is a model for evaluating patient retention across sequential stages of care required to achieve a successful treatment outcome [6]. This model has sequential stages, including awareness, screening, diagnosis, appropriate management, and disease control, that patients navigate while accessing health care services. Acknowledging potential lapses at each stage, the care cascade model identifies critical stages where patients may disengage, hindering them from attaining disease control [7]. The care cascade model was originally conceived for HIV care [8]. It has since been extended to monitor and manage infectious diseases, such as hepatitis C [9] and tuberculosis [6], and has been more recently applied to NCDs [10].

The utility of studying the care cascade of hypertension and diabetes goes beyond the mere exploration of their clinical pathways. It encompasses a broader holistic perspective that includes not only clinical aspects but also the impact on health systems, the quality of life of affected individuals, and the efficiency of health care delivery. Cascade analysis for hypertension and diabetes can help understand the common factors that affect the care model in order to identify appropriate strategies to improve health care for these 2 conditions. However, there is limited evidence synthesis regarding the care cascade of hypertension and diabetes. Therefore, we performed a systematic scoping review with the goal of mapping and describing the current state of evidence on a global scale. We sought to understand the process of hypertension and diabetes management, identify the factors that influence each stage of the care cascade, and explore potential interventions that hold promise for improving care continuity. By synthesizing existing evidence, our findings seek to inform future research endeavors, propelling the advancement of management strategies for hypertension and diabetes.

## Methods

### Study Design

This scoping review was conducted following the stages of a scoping review by Arksey and O’Malley [11] and was reported in accordance with the Preferred Reporting Items for Systematic Reviews and Meta-Analyses extension for Scoping Reviews (PRISMA-ScR) [12].

### Identifying the Research Questions

This scoping review focused on mapping the existing evidence on the care cascade of hypertension and diabetes. The specific research questions were as follows:

1. How the care cascade model has been applied in hypertension and diabetes research?
2. Which stage of the hypertension and diabetes care cascade has the current research in high-income countries (HICs) and LMICs primarily focused on?
3. What are the barriers and facilitators of hypertension and diabetes care cascade completion?
4. What strategies have been employed to improve retention in the hypertension and diabetes care cascade?
5. What are the key knowledge gaps that remain in the literature about the hypertension and diabetes care cascade?

### Identifying Relevant Studies

To identify relevant studies, a systematic search was conducted in MEDLINE, Embase, and Web of Science, using terms pertinent to hypertension, diabetes, and the specific stages of the care cascade. The framework of population, concept, and context was used to identify core concepts related to the research question and inform the search strategy [13]. A complete overview of the search terms for each database is provided in Multimedia Appendix 1. The population of interest in this review was adults aged 18 years or older who had been screened for or diagnosed with hypertension or type 2 diabetes, as well as patients with hypertension or type 2 diabetes undergoing treatment or management. The key concept of the review was the hypertension and diabetes care cascade, with a focus on studies explicitly applying the cascade care model to one or more stages of screening, diagnosis, treatment, and control. Articles describing interventions targeting specific stages of the cascade or factors influencing interventions or outcomes of at least one stage of the care cascade were included. The review aimed to explore a broad range of influencing factors involving both barriers and facilitators, with all pertinent descriptions included, regardless of statistical associations. The contextual scope of this review was in both HICs and LMICs, where hypertension and diabetes care was provided. The timeframe for database searches spanned from January 2011 to January 2023 since the concept of the care cascade was introduced in 2011 [8]. There was no restriction on publication language, allowing for an inclusive evaluation of relevant studies worldwide. The eligibility criteria are shown in Textbox 1.

**Eligibility criteria for screening.**

1. Population: Adults aged 18 years or older who had been screened for or diagnosed with hypertension or type 2 diabetes and patients with known hypertension or type 2 diabetes currently undergoing treatment or management.
2. Concept: Hypertension and diabetes care cascade, with a focus on studies explicitly applying the cascade care model to one or more stages from awareness to control. Interventions that impact patient outcomes and factors that influence implementation outcomes and service outcomes within at least one stage of the care cascade.
3. Context: No limitation. All clinical and primary care settings.
4. Language: No limitation.
5. Published between January 1, 2011, and January 17, 2023.
6. Article type: Original articles and protocol papers, including cross-sectional studies, cohort studies, trials, and implementation studies published in peer-reviewed journals.

### Study Selection

All identified articles were imported into Covidence, and duplicates were removed. Screening proceeded through 2 distinct stages, where titles and abstracts were assessed independently by 4 researchers (JW, FT, XY, and ZW) in pairs, adhering to predefined inclusion and exclusion criteria to determine potential eligibility. In the event of disagreements, a collaborative discussion within the research team swiftly resolved any discrepancies. Subsequently, full-text screening followed a similar process, again involving 4 researchers (JW, FT, YY, and ZW) in pairs. Articles were excluded if they were (1) observing outcomes unrelated to hypertension and type 2 diabetes health care; (2) case reports, conference abstracts, editorials, commentaries, or reviews; and (3) unavailable in full text. Any unresolved discrepancies in article eligibility were resolved by group discussion until a consensus was reached. Notably, to glean insights into ongoing or planned projects and to identify potential interventions and relevant factors, study protocols were intentionally retained and not excluded in the scoping review.

### Charting the Data

A data-charting form was created in Microsoft Excel and pilot tested with 15 articles to establish clarity and consistency in data extraction variables across reviewers. Data extraction was performed by 4 researchers (JW, FT, YY, and ZW). The extracted variables included title, author names, year of publication, study countries, disease of interest (hypertension, diabetes, or both), study method (quantitative, qualitative, or mixed method), study design (cross-sectional study, cohort study, trial, or implementation study), sample size, mean age of participants, stages of the care cascade involved, interventions aimed at improving retention, factors associated with stage completion, and reported outcomes. The care of hypertension and diabetes was divided into multiple stages of the cascade, including awareness, screening, diagnosis, linkage to care, treatment, medication adherence, and ultimately, disease control. The world’s economies were classified based on the World Bank classification as follows: low income, lower-middle income, upper-middle income, and high income [14]. The outcomes were categorized into implementation outcomes, service outcomes, and client outcomes. Implementation outcomes encompassed aspects pertaining to the process of implementing interventions and services for hypertension and diabetes care. This included factors such as acceptability, adoption, appropriateness, cost, feasibility, fidelity, and sustainability of interventions to health care providers or patients. Service outcomes were those related to the quality and effectiveness of the health care services provided to patients with hypertension and diabetes, such as access to health care services, continuity of care, appropriateness of care, equity of service, and health care provider adherence to clinical guidelines. Client outcomes, on the other hand, delved into the impact of health services on patients’ health and clinical conditions, such as blood pressure and blood glucose control, reductions in cardiovascular risk factors, and improvements in overall quality of life. To ensure data accuracy and consistency, a senior researcher (XY) reviewed all extracted data. Any disagreements were resolved by consensus.

### Collating, Summarizing, and Reporting the Results

Interventions and influencing factors were analyzed by cascade stages and focused diseases. Studies that reported multiple stages of the care cascade were included in the synthesis of each relevant stage. The resulting information was subjected to rigorous quantitative analysis, employing frequencies and percentages to elucidate key patterns and trends. Inductive content analysis was performed to generate themes regarding the barriers and facilitators for improving the care cascade in hypertension and diabetes management. The initial list of codes was grouped into categories and then themes against the supporting evidence. Throughout this process, subthemes and themes were discussed and refined within the research team.

### Ethical Considerations

This review does not involve human subject information, primary data collection, or any form of experimentation on individuals.

## Results

### Characteristics of the Included Studies

**Figure 1:**
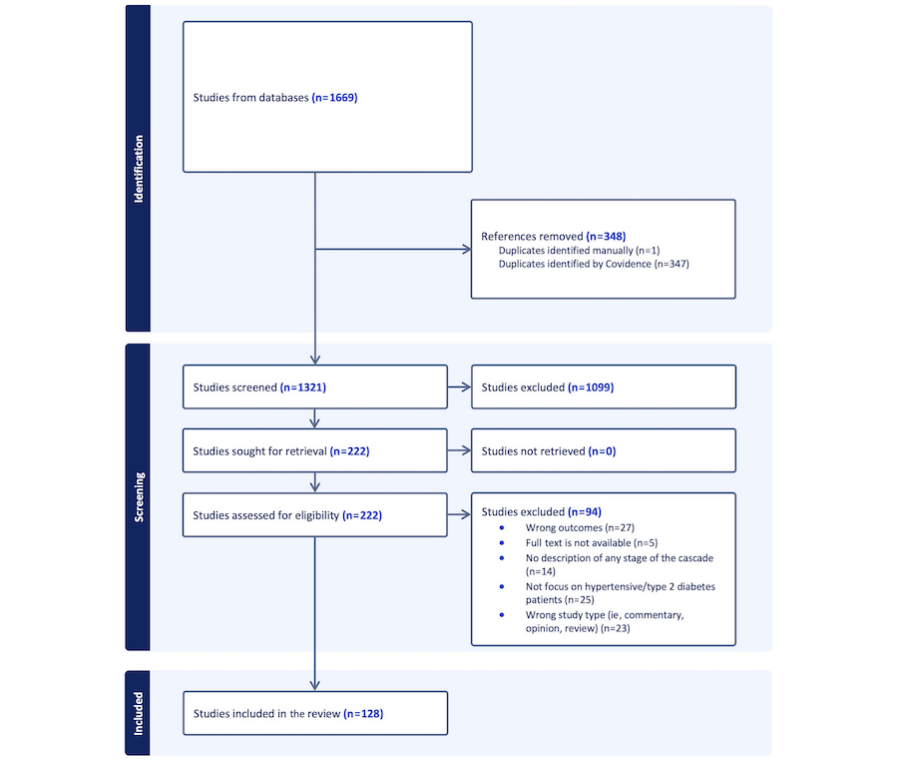
PRISMA (Preferred Reporting Items for Systematic Reviews and Meta-Analyses) flow diagram.

Of the 1321 unique articles identified for the title and abstract screening, 222 were retrieved for full-text review. Of these, 128 were included in the analysis after excluding 94 articles for various reasons (Figure 1).

**Figure 2:**
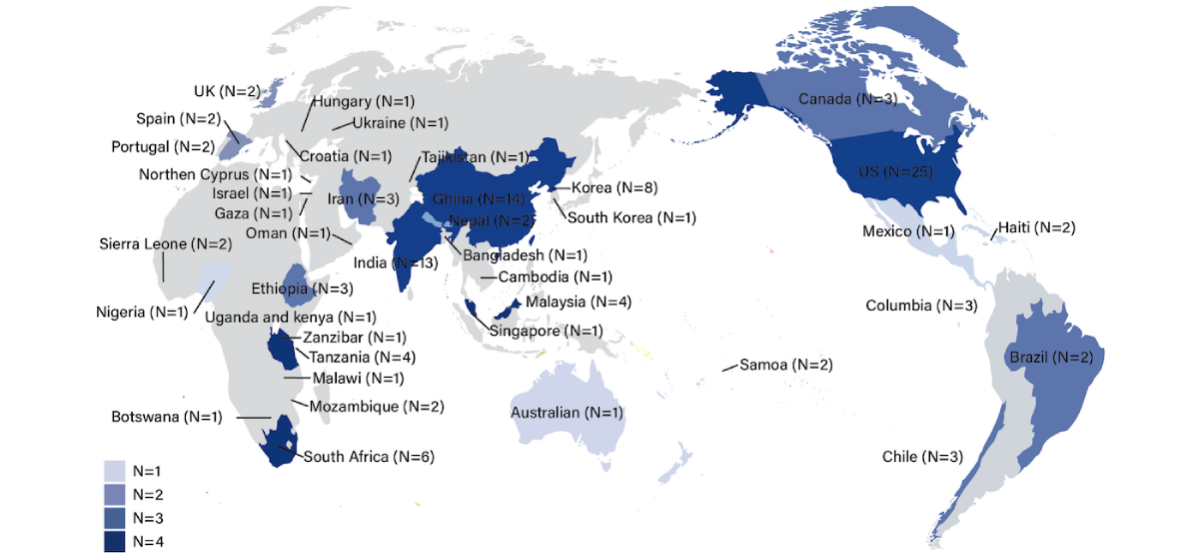
Map of studies included in the scoping review by country (N=40).

The 128 studies originated from 40 countries, with 42.2% (54/128) conducted in HICs (Figure 2). Of the 128 studies, 47 (36.7%) focused on hypertension care, 63 (49.2%) focused on diabetes care, and 18 (14.1%) reported on the care of both diseases. Most studies (104/128, 81.3%) employed quantitative methods. The majority were cross-sectional studies (70/128, 54.7%), followed by cohort studies (26/128, 20.3%). There were 24 (18.8%) trials evaluating interventions to promote retention in at least one cascade stage. Only 8 (6.3%) were implementation studies designed to systematically assess service delivery gaps and identify contextually appropriate solutions to address these bottlenecks. A total of 116 (90.6%) studies reported health receivers’ perspectives, and only 8 (6.3%) studies had health system perspectives. Most studies (83/128, 64.8%) reported client outcomes as primary outcomes, and they mainly focused on the measure of the effectiveness of disease control. Moreover, 16 (12.5%) studies reported service outcomes, and they mainly focused on the measure of satisfaction. Furthermore, 29 (22.7%) studies reported implementation outcomes, such as feasibility, cost, and adoption (Table 1). Detailed characteristics of the 128 included studies are summarized in Multimedia Appendix 2 [10,15-131].

**Table 1:** Summary of the characteristics of the included studies.

Characteristic			Value (N=128), n (%)
**Income level of countries**			
	High income	54 (42.2)	
	Upper-middle income	25 (19.5)	
	Lower-middle income	38 (29.7)	
	Low income	11 (8.6)	
**Disease**			
	Hypertension	47 (36.7)	
	Diabetes	63 (49.2)	
	Hypertension and diabetes	18 (14.1)	
**Study design**			
	Cross-sectional	70 (54.7)	
	Cohort	26 (20.3)	
	Interventional	24 (18.8)	
	Implementation	8 (6.2)	
**Participants**			
	Health receivers	116 (90.6)	
	Health providers	4 (3.1)	
	Health receivers and providers	8 (6.3)	
**Research methods**			
	Quantitative	104 (81.3)	
	Qualitative	11 (8.6)	
	Mixed	13 (10.1)	
**Outcomes**			
	Implementation	29 (22.7)	
	Service	16 (12.5)	
	Client	83 (64.8)	

### Completion of the Hypertension and Diabetes Care Cascade

Only 3 studies documented all 7 cascade stages, with 2 of them focusing on hypertension management and 1 addressing both hypertension and diabetes [15,16,132]. They were all population-based cross-sectional surveys aimed to describe disease prevalence and quantify the unmet need for hypertension and diabetes care. The remaining studies included in our analysis examined specific stages of the care cascade. Among the studies focusing on hypertension, 13 highlighted increasing awareness and knowledge related to hypertension, 8 addressed the importance of screening through blood pressure measurements, 14 focused on the diagnosis of hypertension, 13 explored the linkage to care, 34 discussed the initiation of treatment, 16 emphasized medication adherence, and 26 explored blood pressure management and control. For diabetes care, 8 studies addressed the critical aspect of awareness, 16 concentrated on screening, 20 discussed the diagnosis of diabetes, 21 explored the linkage to care, 38 focused on treatment interventions, 23 examined medication adherence, and 37 investigated the factors impacting diabetes control. In addition, 18 studies adopted an integrated approach, encompassing care for both hypertension and diabetes. Among these studies, 6 addressed awareness, 1 addressed screening, 4 addressed diagnosis, 10 addressed linkage to care, 9 addressed treatment, 2 addressed medication adherence, and 10 addressed control (Table 2).

**Table 2:** Studies across the stages of the care cascade.

Stage of the care cascade	Value, n (%)		
	Hypertension (n=47)	Diabetes (n=63)	Hypertension and diabetes (n=18)
Awareness	13 (27.7)	8 (12.7)	6 (33.3)
Screening	8 (17.0)	16 (25.4)	1 (5.6)
Diagnosis	14 (29.8)	20 (31.7)	4 (22.2)
Linkage to care	13 (27.7)	21 (33.3)	10 (55.6)
Treatment	34 (72.3)	38 (60.3)	9 (50.0)
Medication adherence	16 (34.0)	23 (36.5)	2 (11.1)
Control	26 (55.3)	37 (58.7)	10 (55.6)

### Interventions of the Hypertension and Diabetes Care Cascade

#### Awareness

Various interventions were identified to enhance the knowledge of disease prevention. Health education programs for hypertension and diabetes were emphasized as continuous efforts to support ongoing management and care [17]. The provision of comprehensive education was achieved through training classes and consulting at nutrition-based shared medical appointments [17-19]. Automated outreach call services with the integration of electronic health records emerged as effective approaches [20]. Out-of-hospital continuous nursing interventions and community awareness campaigns were used to augment the awareness [21]. Dissemination of awareness campaign information occurred through various channels, including the internet, public awareness events, and targeted home visits [22].

#### Screening

In the pursuit of enhancing the screening process for hypertension and diabetes, a diverse array of interventions has emerged. The Sustainable East Africa Research in Community Health (SEARCH) study implemented a community health campaign that offered universal adult screening, rendering screening services widely accessible [23]. Moreover, innovative approaches like home-based screening interventions empowered individuals by providing convenience and ease of access to early detection services [24]. Early diabetes detection was prioritized through specialized medical check-ups, facilitating timely intervention [22]. The Integrated Tracking, Referral, and Electronic Decision Support and Care Coordination (I-TREC) program incorporated cutting-edge technologies, including electronic case record forms and clinical decision support systems, streamlining patient information and offering guideline-based screening. Enhanced training for health care providers in NCD management and lifestyle interventions further fortified the screening process [25]. Lastly, efforts were made to strengthen health education and outreach services, particularly targeting individuals without symptoms, to foster a proactive approach to screening [26].

#### Diagnosis

Effective interventions have been deployed to enhance the diagnosis of hypertension and diabetes. Continuous and coordinated care among multi-level health institutions was emphasized to enable timely diagnosis and consistent follow-up for hypertension and diabetes. Telephone peer coaching provided personalized support through weekly calls, aiding in timely diagnosis and empowering patients to engage in self-care [27]. Patient-centered integrated care with advanced technologies, such as electronic case record forms and clinical decision support systems, streamlined patient information and referrals to deliver tailored guideline-based care. Enhanced training for primary health care providers further strengthened timely diagnosis among patients [25].

#### Linkage to Care

Follow-up within 6 weeks at NCD clinics for participants with hypertension, coupled with the use of diabetes self-management record sheets and telephone reinforcement, has shown positive outcomes [133]. Additionally, 8-week training classes encompassing diverse self-care aspects have demonstrated effectiveness [18]. Mobile health applications [28], shared medical appointments [29], telephone peer coaching [27], and regular general practitioner contact [30] have proven to be successful strategies for ensuring a smooth connection to care. Furthermore, providing training in communication skills and self-care education to health providers, along with reduced workload and increased availability of competent diabetes specialist nurses, has contributed to enhancing the linkage to care [31]. Educational group programs, decision support tools, and feedback reports for primary care professionals further reinforced the process [32]. Institution-level continuity of ambulatory care [33], standardized “self-care” programs [22], and patient-held health records [34] have also played pivotal roles in promoting seamless linkage to essential health care services among patients diagnosed with hypertension and diabetes.

#### Treatment

Various interventions have been explored to improve the treatment process for patients with hypertension and diabetes. Lifestyle interventions, including physical activity promotion and heart-healthy diets, have shown promise in improving treatment outcomes [35]. Collaborative care models involving pharmacists and physicians demonstrated positive effects on medication therapy management and overall patient care [36]. Self-monitoring of blood pressure is vital for facilitating appropriate treatment [37]. Additionally, interventions targeting medication affordability [38] and continuity of care [39-41,134] play crucial roles in ensuring optimal treatment adherence. Telehealth and digital interventions, such as continuous remote care and mobile health applications, are being explored for improved treatment accessibility and engagement [28,135]. Integrated care models, employing multidisciplinary teams and decision support tools, have yielded promising outcomes in coordinating comprehensive patient care [20,136]. Targeted education for patients and health care providers can effectively enhance communication and self-care skills [31,32]. Moreover, financial incentive programs, like pay-for-performance schemes, have encouraged optimal health care delivery and reimbursement [42].

#### Medication Adherence

A range of interventions has been investigated to optimize medication adherence in patients with hypertension and diabetes. Community-based interventions with patient education, recall services, and reduced out-of-pocket payments have shown promise in promoting adherence [43]. Self-measured blood pressure monitoring and chronic disease management programs in primary care settings facilitated continuous and comprehensive patient care [38,44]. Telephone peer coaching [27], regular general practitioner contact [30], and continuity of care initiatives [39,40,45,46,134] have also demonstrated positive effects on medication adherence. Collaborative care models, which involve patient-centered coordinated care, referral systems, and diabetes education, have yielded favorable results [47]. Additionally, interventions, such as medication co-payment schemes, enhanced counseling, and training for health care providers in communication skills, have reinforced medication adherence efforts [31,48]. Patient and provider engagement programs, along with pay-for-performance initiatives, have also incentivized optimal medication adherence [32,42]. Integrating pharmacists into multidisciplinary care teams has enhanced medication management and adherence [136].

#### Control

Interventions, including multidisciplinary collaboration, patient education, and technology integration, were adopted to enhance hypertension and diabetes control. The integration of pharmacists into care teams and the transition to specialized diabetes physicians can optimize disease management [49,50,136]. For instance, pharmacist-physician collaborative practice models have shown promise through features like shared medical records, defined interprofessional roles, frequent follow-ups, and collaborative practice agreements [50]. Structured educational programs, both for patients and primary care professionals, offer essential knowledge and support, such as tailored SMS text message communication and telephone peer coaching [27,51]. Patient health records and electronic decision support were used to improve the continuity of care and enable tailored interventions [34]. Additionally, integrated interventions like the EMPOWER-PAR program, grounded in the Chronic Care Model, made contributions to advancing disease control, even in the face of challenges related to health care system constraints [137].

### Barriers and Facilitators of Completing Different Stages of the Care Cascade

In the completion of the hypertension and diabetes care cascade, several barriers and facilitators were identified, encompassing patient-level and system-level factors (Table 3). Patient-level barriers included factors like low socioeconomic status, unhealthy lifestyle choices, and limited health literacy, hindering effective management. Misconceptions about disease and treatment, high treatment costs, and fear of diagnosis also impeded the care progress. At the system level, inadequate resources, heavy workloads, limited capacity in primary care, and a fragmented health system were identified as significant obstacles to effective care.

**Table 3:** Patient-level and system-level barriers and facilitators of completing different stages of the care cascade in both high-income countries and low- and middle-income countries.

Stage and country type		Facilitators	Barriers
**Awareness**			
	HICs^a^	Patient-level: Young age [20,52,132], female sex [52,53], and high socioeconomic status [54,132]	Patient-level: Male sex [55], low income [55,56], poor health literacy [57], minority group [58], and living in resource-limited areas [55]
	LMICs^b^	Patient-level: Aged [53,59], female sex [10,16,59,138], high socioeconomic status [10,16,60], overweight and obesity [59-61], unhealthy lifestyle [59,60], and multimorbidity [61]	Patient-level: Male sex [60], never married [10,60], Hispanic adults [58], and Asian adults [58]
**Screening**			
	HICs	Patient-level: Female sex [62-64], unemployed [61], and single [61]	Patient-level: Low socioeconomic group [15], multimorbidity [65], and minority group [62]
	LMICs	Patient-level: Under the age of 25 years [66] and high socioeconomic group [53,60]; Use of antenatal care services [67]	Patient-level: Low socioeconomic group [16,56], unhealthy lifestyle [66,139], and multimorbidity [65]; Not feeling at risk of hypertension [67]; Not aware of screening services [67]; Low ability to pay for health care [67]; Preference for traditional healers [67]; Perceiving hypertension as a normalized condition [67]
**Diagnosis**			
	HICs	Patient-level: High socioeconomic group [56]	Patient-level: Male sex [68,132], living alone [132], multimorbidity [69], unhealthy lifestyle [132], and living in resource-limited areas [70]
	LMICs	Patient-level: Aged [60,66,71-73], overweight or obesity [63,66,71,74], and presence of other comorbidities [71]	Patient-level: Characteristics of individuals: male sex [64,69,73], low socioeconomic group [72,75], and unhealthy lifestyle [63]; Lack of understanding regarding the importance of following a referral after a positive screening result [76]; Unaffordable health care services [67]; Fear of diagnosis, refusal to accept illness, and noncompliance with referrals [67]; Influence of culture and values, including gender norms [69]; Conflicting time with health facility opening hours [69] System-level: Shortage of physicians [75]; Shortage of health facilities [69,76]; Lack of diagnostic equipment and testing capabilities [26,76]
**Linkage to care**			
	HICs	Patient-level: High socioeconomic status [77] and female sex [77]; Regular clinic visit (due to smoke) [65]; Involvement in other health programs [77] System-level: Presence of a national or local chronic disease management program [78]	Patient-level: Male sex [16,68,77], no health insurance [79], and low education level [64,134]; Presence of other diseases that affect physical activity [134]; Language barriers [31] System-level: Heavy workload affecting patient care [31,77]; Inadequate collaboration among health care team members [31]; Providers’ frustration and aggressive attitudes toward patients [31]
	LMICs	Patient-level: High awareness [80,81]; Social support or involvement of patients’ relatives [81-83]; Context-specific diabetes education and educational materials [84]	Patient-level: Without health insurance [80]; Misconceptions about medications [85]; Cultural beliefs [85] System-level: Absence of guidelines for hypertension and diabetes management [38,44]; Insufficient essential resources and infrastructure [84]
**Treatment**			
	HICs	Patient-level: Young age [20,26,52,72], female sex [10,52,62], White ethnicity [52], and high health literacy [80]; Medicare beneficiary [20] System-level: Presence of a chronic disease management program [44,78]; Home delivery of medication [86]; Good doctor-patient relationship [87,140]	Patient-level: Male sex [68,80], no medical insurance [20,80], low education level [80], unhealthy lifestyle [88], multimorbidity [10,65,80], and language barriers [31]; Psychological fear of treatment [140] System-level: Health care mistreatment attributed to ethnic discrimination [89]; Heavy workload of health providers [34,90,140]; Lack of collaborative strategies among health care teams [140]
	LMICs	Patient-level: High education [55,73], high income [16,55,56,60], and overweight and obesity [59,60,91]; Well-designed education and educational materials [84] System-level: Physician density [75]; Doctors’ interpersonal behaviors and technical competence [92]; Well-trained health workforce [84]	Patient-level: Individual characteristics: never married [60], occupation [66], poor socioeconomic status [15,66,83], poor comprehension [83], unhealthy lifestyle [66], and living in resource-limited areas [16,53,55,64,70]; Misconceptions, lack of confidence, and fear about medications [85,141]; High treatment costs and low ability to pay for medication or transport [26,67]; Poor understanding of asymptomatic conditions requiring treatment [67]; Low risk awareness of nontreatment consequences [67]; Wrong understanding of the disease and its therapy [93]; Lack of social support from peers, family, and the community [93]; High time cost of seeking care [93] System-level: Poor monitoring and lack of a patient follow-up protocol [69,94]; Ineffective medication and physician inertia [69,94]; Ambiguous and inappropriate clinical guidelines in under-resourced areas [93]; Shortage of human resources and equipment for blood pressure monitoring [93]; Limited knowledge and understanding among health care workers [76]; Lack of essential clinical facilities and adequate training of health care workers [76,84]; Absence of organized diabetes services within health care facilities [83]; Rarely receiving feedback on patient management from higher-level facilities [83]
**Medication adherence**			
	HICs	Patient-level: Female sex [80,95], high income [56,95], and high level of hypertension and diabetes knowledge [95,96]; Less negative general beliefs about medications and few concerns about medications [95]	Patient-level: Lower socioeconomic group [95]; Not confident about community pharmacists [141]; Fear about medications [85]; Cultural beliefs influencing management [31]; Multimorbidity [80]; Lack of knowledge leading to misconceptions about disease management [141] System-level: Low primary care visits [80]; Ethnic discrimination in health care settings [89]; Heavy workload [31,90]; Lack of a teamwork approach [31]; Insufficient availability of essential medicines [38]; Ambiguous and inappropriate clinical guidelines [93]
	LMICs	Patient-level: Living in urban areas [16,45,53]	Patient-level: High cost of medication [69]; Personal and cultural beliefs [69]; Wrong understanding of a disease and its therapy among patients [93]; Lack of support from peers, family, providers, and the community [93]
**Control**			
	HICs	Patient-level: Young age [52], female sex [95], high income [74], and being of White ethnicity [52,58]; High level of hypertension and diabetes knowledge [95]; Partner involvement in care [82]; Better self-perceived health status [95]; Healthy lifestyle practices: regular exercise and weight management [97] System-level: Trust between physicians and patients [98]	Patient-level: Characteristics of individuals: male sex [56], ethnic minority [20], and low health literacy [96]; Lack of access to medical care and medications [50]; Using nonoptimal doses of antihypertensive medications [65]; Experiencing adverse events associated with medications [65]
	LMICs	Patient-level: High income [15,54,60,74], older age [19,72], marriage [10,54], fewer complications [65], and health insurance [19,39]; Healthy lifestyle: adopting dietary modifications or engaging in regular exercise [54,99]; Receiving long prescribed medications for hypertension and diabetes [71]; Belief in treatment efficacy and having family support [67]; Timely monitoring of blood pressure and blood glucose [19] System-level: Adequate medications [84]; High physician density [75]	Patient-level: Age ≥75 years [74], male sex [15,55,59,68,100], overweight [59,66,100], low income [55,60,67], specific occupations [66], and low education level [55,68]; Coexisting chronic conditions [68,97]; Living in rural or resource-limited areas [55,60]; Unhealthy lifestyle: smoking and alcohol consumption [66,97,100]; Lack of family/social support [57]; Limited awareness of the lifelong nature of the condition [67]; Complexity of the intervention [68]; Insufficient patient education about the importance of clinical management [67]; Poor communication of treatment monitoring results [67] System-level: Long waiting times at clinics [67]; Negative staff attitudes toward patients [67]; Weak monitoring schedules [67]; Lack of medical resources [84]

^a^HIC: high-income country.

^b^LMIC: low- and middle-income country.

Conversely, various patient-level facilitators positively impacted the cascade. At the patient level, characteristics like high socioeconomic status, positive health behaviors, and strong belief in treatment efficacy played vital roles. Furthermore, timely monitoring of blood pressure and glucose levels, engagement in health programs, and partner involvement were found to be associated with improved outcomes. System-level facilitators included a well-trained health workforce, existing chronic disease management programs, and improved access to medications.

Notably, certain barriers and facilitators were context-specific, with diverse prominence in HICs and LMICs. For instance, lack of understanding and misconceptions were more prevalent in LMICs, while the influence of cultural beliefs and minority status was more pronounced in HICs. Physician density and adequate resources were often noted as facilitators in HICs, while social support and tailored diabetes education were emphasized in LMICs.

## Discussion

This scoping review identified a substantial body of literature investigating the hypertension and diabetes care cascade in both HICs and LMICs. While most studies provided descriptive snapshots of each cascade stage, only a limited number of studies applied implementation cascade analysis to explore the barriers and facilitators of patient retention. Furthermore, there was a paucity of studies evaluating the effects of interventions to bridge gaps between cascade stages. In addition to analyzing the characteristics of the included studies, this scoping review comprehensively summarized key interventions, facilitators, and barriers associated with completing cascade stages. These findings provide critical insights into the existing evidence on hypertension and diabetes management, offering valuable directions for enhancing health care delivery for these chronic conditions.

The results of this scoping review have revealed a notable gap in the existing literature concerning the entire continuum of all stages in the hypertension and diabetes care cascade. The majority of studies predominantly focused on treatment and control for both hypertension and diabetes care. There was a relative paucity of studies examining the stages before treatment initiation despite evidence suggesting that over 50% of patients with hypertension and diabetes who could benefit from treatment never start medication [3,72]. These pretreatment losses accounted for a much greater reduction in effective care than nonadherence to medication [101]. Modeling studies showed that treatment losses earlier on can result in a greater overall reduction in the public health benefit of hypertension management [142,143]. Potential gaps exist in identifying problems and developing strategies to improve awareness, screening, and diagnosis of the 2 diseases. Based on microsimulation modeling, it is estimated that scaling up diagnosis, treatment, and control of diabetes to achieve a hypothetical 80% target for each component of the care cascade would be highly cost-effective [143,144]. Regarding interventions to improve retention across cascade stages, the review emphasizes the importance of awareness campaigns and health education programs to improve patient retention in care and medication adherence. Moreover, interventions targeting the health system (ie, multidiscipline collaborative care, training for primary health care providers, and increasing access to medications) showed promise in improving diagnosis and treatment outcomes. Other innovations in hypertension and diabetes service delivery have been developed and could further enhance quality, but they require further study and proof of effectiveness at scale. Examples include electronic case record–based clinical decision support systems and telephone peer coaching [27,32]. There was a relative dearth of studies incorporating informatics, internet techniques, and mass media to capture public opinions and enhance patient engagement in the management of these conditions. These technologies and communication strategies have only recently gained prominence, and their full potential in the context of hypertension and diabetes care has not yet been comprehensively explored. Our findings parallel another review about the implementation of telemedicine interventions for hypertension and diabetes, indicating that successful implementation of these interventions necessitates comprehensive efforts at all stages of planning, execution, engagement, and reflection and evaluation [145]. The adaptation of interventions to diverse contexts, particularly in LMICs with fragile health systems, warrants future studies. Implementation studies are essential to develop context-specific strategies for incorporating evidence-based interventions effectively into practice [146].

The review also revealed several facilitators and barriers affecting different stages of the care cascade across different income contexts. These insights are of paramount importance, serving as a compass for forthcoming investigations. Future studies can harness these nuanced factors to craft precise context-specific strategies that seamlessly integrate evidence-based interventions into clinical practice. Tailored interventions that address specific patient characteristics, cultural beliefs, and health system constraints are pivotal to enhancing care delivery. The implementation of evidence-based strategies, coupled with the cultivation of patient-centered care, paves the way for health care systems to embark on a journey toward equitable and ameliorated outcomes in hypertension and diabetes management, thereby catering to the unique needs of diverse patient populations.

This review highlights that the provision of integrated care for individuals with both hypertension and diabetes within primary care settings has the potential to be a judicious and efficient approach. The rationale behind this integration lies in the substantial overlap between the risk factors and management pathways of these 2 prevalent chronic conditions. This shared etiological foundation emphasizes the importance of addressing common risk factors, such as dietary patterns, physical activity levels, smoking habits, and weight management, concurrently. By focusing on integrated interventions that aim to modify these shared risk factors, primary care providers can foster holistic and synergistic management. Moreover, primary care providers play a pivotal role in early diagnosis, timely initiation of treatment, and regular follow-up. This proactive approach is essential for mitigating the burden of hypertension and diabetes, as well as their associated complications.

Our study has several strengths. We identified studies from a wide range of geographic and care delivery settings. In addition, this review expands upon the evidence regarding interventions throughout the hypertension and diabetes care cascade, offering insights into diverse strategies to address each stage. By encompassing studies conducted in both HICs and LMICs, this review captures the global perspective on interventions for hypertension and diabetes care. This strengthens the generalizability of the findings and provides insights into the varying challenges and approaches across different health care settings. While this scoping review offers valuable insights into the extensive body of literature concerning the hypertension and diabetes care cascade, it is important to recognize the inherent limitations of this approach compared to systematic reviews and meta-analyses. This breadth of mapping key concepts across diverse domains and disciplines might come at the cost of depth. The interventions described in our review predominantly featured descriptive accounts in the included reports, with the absence of a quantitative assessment of intervention effects, which is important for informing designs in other settings but does not allow for inferences about their effectiveness. As is typical with scoping reviews, we did not assess the quality of the included articles. This inherent limitation underscores the need for further research, particularly systematic reviews and meta-analyses, to delve deeper into the efficacy of interventions across various stages of the hypertension and diabetes care cascade. Moreover, the focus of this review was on studies that explicitly applied the cascade care lens to one or more stages of hypertension and diabetes care, which may have inadvertently excluded studies that explored these critical stages without using the term “cascade” or its associated lexicon. While this search strategy enabled a more targeted examination of research aligned with the cascade model, it also introduced an inadvertent restriction, potentially omitting relevant investigations that did not employ the cascade framework explicitly. This limitation underscores the need for future studies to explore these care stages more comprehensively, even when the cascade terminology is not explicitly invoked, providing a more holistic view of hypertension and diabetes care. Despite this limitation, our scoping review offers valuable insights into a broad landscape of influencing factors and interventions across the care cascade.

In conclusion, this scoping review offers valuable insights into the evidence of the hypertension and diabetes care cascade, highlighting the importance of comprehensive interventions that address all stages of disease management. By identifying facilitators and barriers, the study emphasizes the need for tailored health care strategies to improve patient outcomes. Moving forward, integrating collaborative care models, tailored education programs, and health care system enhancements can potentially enhance disease control and improve the quality of life for individuals living with hypertension and diabetes. These findings have significant implications for clinical practice and health policy, serving as a foundation for future research and efforts to optimize the care cascade for chronic disease management.

## Appendix

Multimedia Appendix 1 Search strategy.

Multimedia Appendix 2 Details of the included studies.

Multimedia Appendix 3 PRISMA-ScR (Preferred Reporting Items for Systematic Reviews and Meta-Analyses extension for Scoping Reviews) checklist.

## Abbreviations

HIC: high-income country
LMIC: low- and middle-income country
NCD: noncommunicable disease

## Data Availability

The data sets generated during or analyzed during this study are available from the corresponding author on reasonable request.
