# Supplementary material for: Understanding Gaps in the Hypertension and Diabetes Care Cascade: Systematic Scoping Review
Source: JMIR Public Health Surveill. 2024 Feb 16;10:e51802. doi: 10.2196/51802 (PMC10907944; doi:10.2196/51802)
Supplement: Multimedia Appendix 1 [file publichealth_v10i1e51802_app1.docx]

**Multimedia Appendix 1.** Search strategy.

| **Database 2011 to January 17, 2023** | **Search Strategies** |
| --- | --- |
| Medline, Embase | 1. (cascade* adj2 care).ti,ab,kw.  2. (continu* adj2 care).ti,ab,kw.  3. ('control cascade' or 'treatment cascade' or 'prevention cascade' or 'control continuum' or 'treatment continuum' or 'prevention continuum').ti,ab,kw.  4. 1 or 2 or 3  5. (screen* or examin*).ti,ab,kw.  6. diagnos*.ti,ab,kw.  7.(treat* or manage* or intervent* or medication or therapy).ti,ab,kw.  8. ('follow up' or 'follow-up' or 'drop out' or 'drop-out' or 'drop off' or 'drop-off').ti,ab,kw.  9. (adherence or nonadherence or compliance or noncompliance or completion or compliant or noncompliant).ti,ab,kw.  10. 5 or 6 or 7 or 8 or 9  11. 4 and 10  12. exp hypertension/  13. (hypertens* or prehypertens*).ti,ab,kw.  14. exp blood pressure/  15. ((elevat* or increase* or lower or high or rais* or rising) adj (arterial pressure or blood pressure or diastolic blood pressure or systolic blood pressure)).ti,ab.  16. Or/12-15  17. exp Diabetes Mellitus, Type 2/  18. (MODY or NIDDM or T2DM or T2D).ti,ab,kw.  19. (non insulin* depend* or noninsulin* depend* or noninsulin?depend* or non insulin?depend*).ti,ab,kw.  20. ((typ? 2 or typ? II or typ?2 or typ?II) adj2 diabet*).ti,ab,kw.  21. (((late or adult* or matur* or slow or stabl*) adj3 onset) and diabet*).ti,ab,kw.  22. or/17-21  23. 16 or 22  24. 11 and 23  25. (exp animal/ or animal.hw. or nonhuman/) not (exp human/ or human cell/ or (human or humans).ti.)  26. 24 not 25  27. limit 26 to yr="2011 -Current" |
| Web of Science | 1: ((TI=(cascade* near/2 care)) OR AB=(cascade* near/2 care)) OR AK=(cascade* near/2 care)  2: ((TI=(continu* near/2 care)) OR AB=(continu* near/2 care)) OR AK=(continu* near/2 care)  3: ((AK=('control cascade' or 'treatment cascade' or 'prevention cascade' or 'control continuum' or 'treatment continuum' or 'prevention continuum')) OR TI=('control cascade' or 'treatment cascade' or 'prevention cascade' or 'control continuum' or 'treatment continuum' or 'prevention continuum')) AND AB=('control cascade' or 'treatment cascade' or 'prevention cascade' or 'control continuum' or 'treatment continuum' or 'prevention continuum')  4: #1 OR #2 OR #3  5: ((AK=(screen* or examin*)) OR TI=(screen* or examin*)) OR AB=(screen* or examin*)  6: ((AK=(diagnos*)) OR TI=(diagnos*)) OR AB=(diagnos*)  7: ((AK=(treat* or manage* or intervent* or medication or therapy)) OR TI=(treat* or manage* or intervent* or medication or therapy)) OR AB=(treat* or manage* or intervent* or medication or therapy)  8: ((AK=('follow up' or 'follow-up' or 'drop out' or 'drop-out' or 'drop off' or 'drop-off')) OR TI=('follow up' or 'follow-up' or 'drop out' or 'drop-out' or 'drop off' or 'drop-off')) OR AB=('follow up' or 'follow-up' or 'drop out' or 'drop-out' or 'drop off' or 'drop-off')  9: ((AK=(adherence or nonadherence or compliance or noncompliance or completion or compliant or noncompliant)) OR TI=(adherence or nonadherence or compliance or noncompliance or completion or compliant or noncompliant)) OR AB=(adherence or nonadherence or compliance or noncompliance or completion or compliant or noncompliant)  10: #5 OR #6 OR #7 OR #8 OR #9  11: #4 AND #10  12: TS=(hypertension)  13: ((TI=(hypertens* or prehypertens)) OR AB=(hypertens* or prehypertens)) OR AK=(hypertens* or prehypertens)  14: TS=(blood pressure)  15: (TI=((elevat* or increase* or lower or high or rais* or rising) near ("arterial pressure" or "blood pressure" or "diastolic blood pressure" or "systolic blood pressure"))) OR AB=((elevat* or increase* or lower or high or rais* or rising) near ("arterial pressure" or "blood pressure" or "diastolic blood pressure" or "systolic blood pressure"))  16: #12 OR #13 OR #14 OR #15  17: TS=(Diabetes Mellitus, Type 2)  18: ((TI=(MODY or NIDDM or T2DM or T2D)) OR AB=(MODY or NIDDM or T2DM or T2D)) OR AK=(MODY or NIDDM or T2DM or T2D)  19: ((TI=(non insulin* depend* or noninsulin* depend* or noninsulin?depend* or non insulin?depend*)) OR AB=(non insulin* depend* or noninsulin* depend* or noninsulin?depend* or non insulin?depend*)) OR AK=(non insulin* depend* or noninsulin* depend* or noninsulin?depend* or non insulin?depend*)  20: ((TI=(("typ? 2" or "typ? II" or "typ?2" or "typ?II") near/2 diabet*)) OR AB=(("typ? 2" or "typ? II" or "typ?2" or "typ?II") near/2 diabet*)) OR AK=(("typ? 2" or "typ? II" or "typ?2" or "typ?II") near/2 diabet*)  21: ((TI=(((late or adult* or matur* or slow or stabl*) near/3 onset) and diabet*)) OR AB=(((late or adult* or matur* or slow or stabl*) near/3 onset) and diabet*)) OR AK=(((late or adult* or matur* or slow or stabl*) near/3 onset) and diabet*)  22: #17 OR #18 OR #19 OR #20 OR #21  23: #16 OR #22  24: #11 AND #23  25: ((TI=(animal)) OR TI=(nonhuman)) not TI=(human or human cell or(human or humans))  26: #11 AND #23 NOT ((TI=(animal)) OR TI=(nonhuman)) not TI=(human or human cell or(human or humans))  27: #11 AND #23 NOT ((TI=(animal)) OR TI=(nonhuman)) not TI=(human or human cell or(human or humans)) and PY=(2011-2023) |
