# Supplementary material for: Understanding Gaps in the Hypertension and Diabetes Care Cascade: Systematic Scoping Review
Source: JMIR Public Health Surveill. 2024 Feb 16;10:e51802. doi: 10.2196/51802 (PMC10907944; doi:10.2196/51802)
Supplement: Multimedia Appendix 2 [file publichealth_v10i1e51802_app2.docx]

**Multimedia Appendix 2.** Details of the included studies.

| Author, Year | Country | Disease focused | Participants | Study design | Methods | Cascade stage reported | Outcomes | Interventions for improving the retention of patients | Barriers associated with patient retention | Facilitators associated with patient retention |
| --- | --- | --- | --- | --- | --- | --- | --- | --- | --- | --- |
| Sisson et al. 2016 | US | HTN | Health receivers | Cohort | Quantitative method | Treatment, control | The change in mean systolic and diastolic BP from baseline to the last measured BP in 2013 | Pharmacist involvement, pharmacist-physician collaborative practice model (PPCPM) | Lack of access to medical care and medications |  |
| Tang et al. 2017 | China | HTN | Health receivers | Interventional study | Quantitative method | Link to care | SBP and health-related quality of life measured by SF36 | Integrated care model, financial incentive |  |  |
| Carrillo-Larco et al. 2022 | 191 LMICs | HTN | Health receivers and providers | Cross sectional study | Quantitative method | Diagnosis, treatment, control | Prevalence of hypertension |  | Without enough physicians, low income | Physician density |
| Geraedts et al. 2021 | Sierra Leone | HTN | Health receivers | Cross sectional study | Quantitative method | Diagnosis, treatment, medication adherence | The number of patients | Invest in the training of community health officers, increasing awareness, risk factor screening for early diagnosis | Maternal and child healthcare services and gender norms, rural, fewer health facilities, poor adherence to the prescribed medication, poor monitoring, costs, personal and cultural beliefs |  |
| Kim et al. 2016 | Korea | HTN | Health receivers | Cohort | Quantitative method | Treatment | Most Frequent Provider Continuity (MFPC), Modified Continuity Index (MMCI), and Continuity of Care (COC) |  | Women, low ambulatory care visits, high number of providers, main medical institution (specialized general hospitals), and a high Charlson's comorbidity score | Public centers |
| Londoño Agudelo et al. 2021 | Colombia | HTN | Health receivers | Cross sectional study | Quantitative method | Awareness, screening, diagnosis, linked to care, treatment, medication adherence, control | BP |  | Young, men, living alone, unhealthy lifestyle | High education |
| Foti et al. 2019 | US | HTN | Health receivers | Cross sectional study | Quantitative method | Awareness, treatment, control | BP |  | Younger men, older women, blacks | Younger women, older men, whites |
| Passi-Solar et al. 2020 | Chile | HTN | Health receivers | Cross sectional study | Quantitative method | Awareness, screening, diagnosis, treatment, medication adherence, control | BP |  | Low income |  |
| Chikafu et al. 2021 | South Africa | HTN | Health receivers | Cross sectional study | Quantitative method | Awareness, screening, diagnosis, treatment, control | SBP, DBP |  | Employed, in a cohabiting union | Overweight and obesity, unemployed, single, female |
| Metz et al. 2022 | Haiti | HTN | Health receivers | Cross sectional study | Quantitative method | Screening, diagnosis, treatment, medication adherence, control | SBP, DBP | Improve medication access, affordability, and adherence |  | Obesity, high education |
| Wozniak et al. 2016 | US | HTN | Health receivers | Cross sectional study | Quantitative method | Awareness, treatment, control | BP | Automated outreach call services, hypertension awareness campaigns, use electronic health record (EHR) | Elderly and minorities | Medicare beneficiaries |
| Dhungana et al. 2022 | Nepal | HTN | Health receivers | Cross sectional study | Quantitative method | Awareness, screening, treatment, control | Prevalence of hypertension screening, awareness, treatment, and control |  | Men, never married, students, low education, low income, without health insurance | Wealth, healthy lifestyle, overweight and obese, multimorbidity |
| Zhao et al. 2021 | China | HTN | Health receivers | Cross sectional study | Quantitative method | Diagnosis, treatment, control | BP |  | Living in resource-limited areas, aged 75+ | High income, obese |
| Abu Hamad et al. 2023 | Gaza | HTN | Health receivers | Cross sectional study | Quantitative method | Diagnosis, treatment, control | Prevalence of hypertension diagnosis and control |  |  | Old, refugees, ex-smokers, overweight or obese, multimorbidity, female, healthy lifestyle |
| Lee et al. 2022 | Korea | HTN | Health receivers | Cross sectional study | Quantitative method | Diagnosis | Number of Visits, Most Frequent Provider Continuity (MFPC) |  | Social distancing and isolation |  |
| Heller et al. 2020 | Uganda and Kenya | HTN | Health receivers | Interventional study | Quantitative method | Screening, diagnosis, linked to care, control | Effectiveness in linking to care and achieving HTN control | The Sustainable East Africa Research in Community Health (SEARCH) study |  |  |
| Amarchand et al. 2023 | India | HTN | Health receivers | Cross sectional study | Quantitative method | Awareness, screening, diagnosis, treatment, medication adherence, control | BP |  | Living in resource-limited areas | Age group 50-69 years, women, high income, high education, not working, professionals |
| Meador et al. 2022 | US | HTN | Health receivers | Cross sectional study | Quantitative method | Treatment, control | The number of patients with hypertension | Conjunction with co-interventions (medication titration, therapeutic lifestyle changes, and education and counseling) |  | Telehealth |
| Shamsi et al. 2021 | Iran | HTN | Health receivers | Interventional study | Quantitative method | Treatment, control | SBP, DBP | A healthy lifestyle, 12 consultation sessions, the PA courses |  |  |
| Fraser-Hurt et al. 2022 | Samoa | HTN | Health receivers | Cross sectional study | Mixed methods | Screening, diagnosis, treatment, control | Hypertension prevalence |  | Not feeling at risk of hypertension, not aware of screening service, low ability to pay for health care, preference for traditional healers, perceiving hypertension as a normalized condition, fear of diagnosis, refusal to accept illness, and non-compliance with referrals, low ability to pay for health care, poor understanding, long waiting times at clinics, negative staff attitudes towards patients, weak monitoring schedules | Use of antenatal care services, Understanding the importance of following a referral after a positive screen, belief that medication works, family support |
| Maroof et al. 2022 | India | HTN | Health receivers | Cross sectional study | Mixed methods | Control | Prevalence of hypertension control |  | 40-49 years, male, Sikhs and Jain, obese, alcoholics |  |
| Hanafi et al. 2015 | Malaysia | HTN | Health receivers | Cohort | Quantitative method | Linked to care, control | BP |  |  | Personal continuity was not associated with BP control. |
| Tapela et al. 2020 | Botswana | HTN | Health receivers | Cross sectional study | Quantitative method | Awareness, treatment, control | Hypertension prevalence |  | Male, obese | Old, female, receipt of lifestyle risk factor, multimorbidity |
| Shah et al. 2022 | Malaysia | HTN | Health receivers | Cross sectional study | Mixed methods | Screening, diagnosis, linked to care, treatment, control | The prevalence of unmet need for hypertension care |  | Multimorbidity, using nonoptimal doses of antihypertensive medication, experiencing adverse events associated with medications, polypharmacy | Regular clinic visit (due to smoke) |
| Macinko et al. 2018 | Brazil | HTN | Health receivers | Cross sectional study | Mixed methods | Diagnosis, linked to care, treatment, control | The prevalence of hypertension control |  | Male, low education, another chronic condition, the complexity of lifestyle modification | Age, private health plan |
| Hirsch et al. 2014 | US | HTN | Health receivers | Interventional study | Quantitative method | Treatment | Mean reduction in SBP | Pharmacist-physician collaborative MTM (PharmD-PCP MTM) |  |  |
| Kunwar et al. 2021 | India | HTN | Health receivers | Cross sectional study | Quantitative method | Control | The feasibility of community-level drug distribution for patients with hypertension | Community distribution of medications |  |  |
| Son et al. 2019 | Korea | HTN | Health receivers | Interventional study | Quantitative method | Treatment, medication adherence | Insurance benefit days based on year and region, dispensation per prescription (DPP), and dispensation days per patient (DDPP) with hypertension as proxy indicators | A community-based intervention (patient education, recall and remind service, and reduction of out-of-pocket payment) |  |  |
| Qiu et al. 2019 | China | HTN | Health receivers | Cross sectional study | Quantitative method | Linked to care, treatment, medication adherence | Continuity of care for hypertensive patients |  | Male, without medical insurance, low education, underwent family visits less than 3 times per year, multimorbidity | Good general health perception |
| Adinkrah et al. 2020 | US | HTN | Health receivers | Cross sectional study | Quantitative method | Medication adherence, control | Adherence to medication and lifestyle recommendations |  |  | Women, less financial strain, a higher level of continuity of medical care, less negative general beliefs about medications, fewer concerns about antihypertensive medications, and a higher level of hypertension knowledge |
| Roy et al. 2021 | US | HTN | Health receivers | Cohort | Mixed methods | Diagnosis, treatment, medication adherence | Engagement with blood pressure management | Self-measured blood pressure monitoring (SMBP) |  |  |
| Bay et al. 2019 | Mozambique | HTN | Health receivers | Cohort | Quantitative method | Diagnosis, treatment, medication adherence | Patient' s flow and care and health facility’s infrastructure and resources |  | Insufficient availability of diagnostics, insufficient availability of essential medicines low affordability of the families |  |
| Onwudiwe et al. 2011 | US | HTN | Health receivers | Cross sectional study | Quantitative method | Medication adherence, control | Self-management of diabetes |  | Lack of knowledge of target blood glucose and blood pressure, inadequate health literacy |  |
| Goudge et al. 2018 | South Africa | HTN | Health receivers | Interventional study | Quantitative method | Linked to care | The change in the difference of percentage of clinic users | Two LHWs (lay health workers) assigned to each clinic |  |  |
| Chukwuma et al. 2019 | Tajikistan | HTN | Health receivers | Implementation study | Mixed methods | Diagnosis, treatment, medication adherence | Prevelence of hypertension |  | Wrong understandings, high time and monetary costs of seeking care, ambiguous and inappropriate clinical guidelines, shortages of human resource for health and equipment for blood pressure monitoring, a lack of support from peers, family, providers, and the community for initiation and adherence to hypertension care |  |
| Lee et al. 2022 | South Korea | HTN | Health receivers | Interventional study | Quantitative method | Linked to care, treatment, medication adherence | Continuity of care, sequential continuity of care (SECON), usual provider care (UPC), integrated continuity of care (ICOC) |  |  | A chronic disease management program |
| Barrera et al. 2021 | Colombian | HTN | Health receivers | Cohort | Quantitative method | Treatment, medication adherence, control | Controlled HPB defined as BP <140/90 mmHg | Continuity of care |  | Primary health care-insurance plans, contributive and subsidized |
| Choi et al. 2020 | Korea | HTN | Health receivers | Cohort | Quantitative method | Treatment, medication adherence | The risk of CVD, CHD, and stroke, hypertensive medication compliance | Continuity of care (COC) |  |  |
| Geldsetzer et al. 2022 | 44 low-income and middle-income countries | HTN | Health receivers | Cross sectional study | Quantitative method | Screening, diagnosis, treatment, control | Prevalence of hypertension |  |  | Women, old, high education, high income |
| Obagha et al. 2022 [16] | Nigeria | HTN | Health receivers | Cross sectional study | Quantitative method | Screening, diagnosis, treatment, control | Prevalence of hypertension | Hypertension care cascade (HCC) |  |  |
| Isangula et al. 2020 | Tanzania | HTN | Health receivers | Cross sectional study | Quantitative method | Treatment | Doctors' trustworthiness |  |  | Doctors' interpersonal behaviors and technical competence |
| Kothavale et al. 2022 | India | HTN | Health receivers | Cohort | Quantitative method | Awareness, screening, diagnosis, treatment, control | BP |  | Caste, religion, living arrangement, MPCE quintile, residence, family history of hypertension, working status, and alcohol consumption |  |
| Berry et al. 2017 | South Africa | HTN | Health receivers | Cross sectional study | Quantitative method | Screening, diagnosis, treatment, control | Prevalence of hypertension |  | Asian/Indian/other subpopulation | Women, white |
| Aggarwal et al. 2021 | US | HTN | Health receivers | Cross sectional study | Quantitative method | Awareness, treatment, control | Prevalence of hypertension awareness, treatment, and control |  | Black adults, Asian adults | White adults |
| Kothavale et al. 2022 | India | HTN | Health receivers | Cross sectional study | Quantitative method | Screening, diagnosis, treatment, control | BP |  | Rural, age, obesity, alcohol consumption, occupation | Urban, high income |
| Chham et al. 2022 | Cambodia | HTN | Health receivers | Cross sectional study | Quantitative method | Awareness, screening, diagnosis, linked to care, treatment, medication adherence, control | Prevalence of hypertension, BP |  | Men, inefficiency of care and treatment | High income |
| Sudharsanan et al. 2020 | South Africa | HTN | Health receivers | Cross sectional study | Quantitative method | Screening, control | SBP, DBP | Home-based screening intervention |  |  |
| Olry de Labry Lima et al. 2017 | Spain | T2DM | Health receivers | Interventional study | Quantitative method | Linked to care | Difference in HbA1c after 12 months | Use a diabetes self-management record sheet (DSMRS) |  |  |
| Kargar et al. 2014 | Iran | T2DM | Health receivers | Interventional study | Quantitative method | Awareness, linked to care | QOL score(quality of life outcomes) | Training classes, 8-week training classes |  |  |
| Cole­ et al. 2013 | US | T2DM | Health receivers | Interventional study | Quantitative method | Awareness, linked to care | FBG | Three 90-minute nutrition SMA (SMA is nutrition-based shared medical appointment) |  |  |
| Wang et al. 2019 | China | T2DM | Health receivers | Interventional study | Quantitative method | Linked to care | FPG, self-management ability, rehospitalization rate and number of hospital visits | Use mobile health application |  |  |
| Halalau et al. 2022 | US | T2DM | Health receivers | Interventional study | Quantitative method | Treatment, control | HbA1c | A pharmacist-managed diabetes clinic |  |  |
| Kazemian et al. 2019 | US | T2DM | Health receivers | Cross sectional study | Quantitative method | Diagnosis, linked to care, treatment, control | HbA1c, BP, LDL-C |  | Women, age, smoking, high drug costs |  |
| Yen et al. 2016 | China | T2DM | Health receivers | Cohort | Quantitative method | Link to care, control | P4P program enrollees | Pay-for-performance (P4P) program | Male, age<35, urban, greater severity of diabetes complications, older age of the main physician, high service volume, having a regional or private hospital as the main healthcare organization, and change of physician | Female, aged over 35, high income, better health status, high physician service volume, long-term relationship between a patient and a physician |
| Chen et al. 2022 | China | T2DM | Health receivers | Cross sectional study | Quantitative method | Awareness, control | HbA1c |  | Single, low education | BMI, age, without religious beliefs, high income, married, the number of chronic diseases and complications, exercise |
| Fung et al. 2015 | China | T2DM | Health receivers | Cohort | Quantitative method | Treatment, control | HbA1c |  | Refuse insulin injection | Use metformin, exercise, body weight management, regular monitoring and early intervention |
| Jalilian et al. 2021 | Iran | T2DM | Health receivers | Cross sectional study | Quantitative method | Link to care, treatment | Likert scale score |  | Financial barriers (resulting from disease treatment costs) | Quality of care, accessibility, awareness and attitude, social support |
| Manne-Goehler et al. 2019 | 28 LMICs | T2DM | Health receivers and providers | Cross sectional study | Quantitative method | Awareness, screening, diagnosis, linked to care, treatment, control | HbA1c |  |  | old, educational attainment, BMI, upper-middle income countries |
| Ortiz et al. 2016 | Chile | T2DM | Health receivers | Cross sectional study | Quantitative method | Link to care, treatment, medication adherence | HbA1c |  | Healthcare mistreatment (attributed to ethnic discrimination) |  |
| Dedefo et al. 2020 | Ethiopia | T2DM | Health receivers | Cross sectional study | Quantitative method | Awareness, treatment, control | FBG |  | Poor knowledge of diabetes, duration of diabetes of >10 years, taking insulin alone and taking metformin plus glibenclamide, unemployment, lack of family/social support |  |
| Doubova et al. 2018 | Mexico | T2DM | Health receivers | Cohort | Quantitative method | Link to care, medication adherence, control | The quality of the process of healthcare (QPHC) and the outcomes of healthcare (QOHC) |  | Loss of job-related right to healthcare | Continuous healthcare |
| Heisler et al. 2021 | US | T2DM | Health receivers | Interventional study | Quantitative method | Link to care, treatment, control | HbA1c | Shared Medical Appointments (SMAs) |  |  |
| Holman et al. 2022 | UK | T2DM | Health receivers | Cohort | Quantitative method | Link to care, control | HbA1c |  | In remission from type 2 diabetes |  |
| Soriano et al. 2022 | US | T2DM | Health receivers | Cross sectional study | Quantitative method | Link to care, control | Glucose level |  |  | Momentary partner involvement in diabetes self-care |
| Thomas et al. 2022 | US | T2DM | Health receivers | Cohort | Quantitative method | Link to care, control | HbA1c |  | Changes in address |  |
| Wang et al. 2020 | China | T2DM | Health receivers | Interventional study | Quantitative method | Awareness, control | FBG, HbA1c | Out-of-hospital continuous nursing intervention |  | Age, medical insurance, income, timely monitoring of blood glucose |
| Chalermsri et al. 2014 | Thailand | T2DM | Health receivers | Interventional study | Quantitative method | Screen, diagnosis, link to care, treatment, control | Clinical outcomes, healthcare maintenance | Continuity of care clinic |  |  |
| Reutens et al. 2012 | Beijing, Hong Kong, Seoul, Hanoi, Manila, Taipei, Bangkok, Surabaya, Singapore and Johor Bahru) | T2DM | Health receivers and providers | Interventional study | Quantitative method | Treatment, control | HbA1c | A structured GP education program |  |  |
| McGowan et al. 2019 | Columbia | T2DM | Health receivers | Interventional study | Quantitative method | Diagnosis, link to care, treatment, medication adherence, control | Glycated hemoglobin, self-reported health, fatigue and pain, activation, empowerment, self-efficacy, depression, communication with physician | Telephone Peer Coaching |  |  |
| Senteio et al. 2021 | US | T2DM | Health receivers | Cross sectional study | Quantitative method | Control | Poor diabetes control based on HbA1c |  | Women, rural areas, having asthma decreases |  |
| Mayer et al. 2021 | US | T2DM | Health receivers | Cross sectional study | Quantitative method | Link to care, treatment | Outpatient utilization, Medicaid enrollment, transitions in care measures, Bice-Boxerman COC Index, Usual Provider of Care (UPC) Index | New York State's Health Homes program |  | Medicaid enrollment, |
| Lustman et al. 2016 | Israel | T2DM | Health receivers | Cohort | Quantitative method | Control | HB1Ac, blood pressure, hospitalization, mortality | Interpersonal continuity |  |  |
| Malcolm et al. 2013 | Canada | T2DM | Health receivers | Cross sectional study | Quantitative method | Medication adherence | Medication use, processes of care indicators, outcome of care indicators, number of visits to each diabetes team member (physician, nurse, dietician, social worker), and current status with FEDC (discharged back to PCP, retained, or defaulted) |  | Patients have perceived their diabetes as “less important” relative to their other medical problems； Primary care physicians may have been unaware that the patient had stopped attending diabetes clinic visits. |  |
| Athinarayanan et al. 2019 | US | T2DM | Health receivers | Interventional study | Quantitative method | Treatment, | Clinical markers of diabetes and cardiometabolic health | Digitally-monitored continuous care intervention | Intervening life events , difficulty attending or completing laboratory and clinic visits, insufficient motivation for participation in the intervention |  |
| David et al. 2022 | South Africa | T2DM | Health receivers | Cross sectional study | Mixed methods | Treatment, medication adherence | HbA1c | Home delivery of medication | Safety concerns, transportation challenges, PCF overcrowding and long waiting times |  |
| Smith et al. 2015 | US | T2DM | Health receivers | Cross sectional study | Quantitative method | Screen, diagnosis | Rates of new DM-II diagnoses and HbA1c screening | Patient-centered medical home |  |  |
| Xu et al. 2020 | Nepal | T2DM | Health receivers | Implementation study | Quantitative method | Awareness, screen, diagnosis | HbA1c, RE-AIM (reach, effectiveness, adoption, implementation and maintenance) | Community awareness campaigns, nurse-led continuum of care for people with diabetes and prediabetes, training of nurses, screening programs, linkage to clinical care, community follow-up counseling and support for the diabetic patients, prevention programs for prediabetic participants |  |  |
| Lewis and Newell 2014 | Bangladesh | T2DM | Health receivers and providers | Cross sectional study | Quantitative method | Diagnosis, treatment, medication adherence | Patients' perspectives of care for type 2 diabetes | Specialist counselling, packages of low cost and comprehensive treatments | Away from specialist centers, limited knowledge and understanding of diabetes, availability and costs of services, lack of essential clinical facilities and adequate training of healthcare workers |  |
| Dambha-Miller et al. 2018 | UK | T2DM | Health receivers | Cross sectional study | Mixed methods | Treatment | Patients' views on interactions with practitioners for type 2 diabetes |  |  | Face-to-face contact with practitioner, length of patient-practitioner interaction, continuity of care |
| Mwangome et al. 2017 | Tanzania | T2DM | Health providers | Cross sectional study | Quantitative method | Link to care, treatment | Perceptions on diabetes care provision among health providers |  | Providers perceived no obligation to provide diabetes care, not systematically recorded, no organization of diabetes services, rarely received feedback on patient management from the higher-level facility, patients’ poor socioeconomic status, poor comprehension, long distances to the health facilities, ignore lifestyle advice, use of traditional medicines | Involvement of patients’ relatives in patient care |
| Jermendy et al. 2012 | Hungary | T2DM | Health receivers | Interventional study | Quantitative method | Treatment | Length of therapy | Metformin and/or sulphonylureas | Sulphonylurea monotherapy | Lower doses and smaller boxes |
| Mathew et al. 2022 | Singapore | T2DM | Health receivers | Cross sectional study | Quantitative method | Treatment | The key aspect of the patient-provider relationship that affects the initial insulin acceptance and continued adherence |  |  | Trusting relationship, good communication skills and patient-centered decision |
| Hong et al. 2013 | Korea | T2DM | Health receivers | Cohort | Quantitative method | Treatment | Hospitalization, mortality and healthcare costs | Continuity of ambulatory care |  |  |
| Jug et al. 2022 | Croatia | T2DM | Health receivers | Cohort | Quantitative method | Treatment | HbA1c, LDL, eGFR, blood pressure, BMI, eye fundus and neurological findings, number of check-ups and vaccination against the flu | Family medicine specialists |  |  |
| Pan et al. 2017 | China | T2DM | Health receivers | Cohort | Quantitative method | Treatment, control | Survival status and physician continuity | Pay-for-Performance Programs |  | Trust between physicians and patients |
| Ali et al. 2014 | US | T2DM | Health receivers | Cross sectional study | Quantitative method | Awareness, diagnosis, link to care, treatment, medication adherence, control | Prevelence of diabetes |  |  |  |
| Youens et al. 2021 | Australian | T2DM | Health receivers | Cohort | Quantitative method | Link to care, treatment, medication adherence | HbA1c, estimated glomerular filtration rate | Regularity/continuity of GP contact |  |  |
| Madede et al. 2022 [138] | Mozambique | T2DM | Health receivers | Cross sectional study | Quantitative method | Awareness | Prevelence of diabetes |  |  |  |
| Chen and Cheng 2016 | China | T2DM | Health receivers | Cohort | Quantitative method | Medication adherence | Medication possession ratio | Continuity of care |  | older, female, urban |
| Chen et al. 2013 | China | T2DM | Health receivers | Cross sectional study | Quantitative method | Medication adherence | Medication possession ratio, hospitalized or had an ED visit for diabetes or cardiovascular/cerebrovascular conditions | Continuity of care |  |  |
| Gomes et al. 2017 | Brazil | T2DM | Health receivers | Interventional study | Quantitative method | Control | Blood pressure, BMI, WC, HbA1c, laboratory data (FPG, total cholesterol, HDL, LDL, triglycerides, urea, creatinine), changes in medications | Have a family caregiver, and the education provided through telephone calls to patients' family members and caregivers |  |  |
| Desse et al. 2022 | Ethiopia | T2DM | Health receivers and providers | Cross sectional study | Quantitative method | Link to care, treatment, control | Perspectives of patients, health professionals, and policymakers on current practices and their future preferences for type 2 diabetes care in a tertiary hospital |  | Lacked essential resources and infrastructure, lack of comprehensive diabetes treatment approaches | Laboratory and diagnostic tests, medications, adequate physical spaces, a trained health workforce, diabetes education and educational materials |
| Desse et al. 2022 | Ethiopia | T2DM | Health receivers and providers | Cross sectional study | Quantitative method | Link to care, treatment, medication adherence, control | Gaps in diabetes management | VICKY: patient-centered collaborative care, referral system, collaborative care and documentation of care, diabetes education and counselling, educational materials | Lack of structured type 2 diabetes education, counselling, and collaborative care of type 2 diabetes, |  |
| Stuart et al. 2020 | Ukraine | T2DM | Health receivers | Cross sectional study | Quantitative method | Screen, diagnosis, treatment, medication adherence, control | Prevelence of diabetes | Outreach/community-based screening, medication copayment schemes, enhanced adherence counseling |  |  |
| Yan et al. 2021 | Canada | T2DM | Health receivers | Cohort | Quantitative method | Treatment | Recurrent hyperglycemia ED visit, hospitalization for hyperglycemia | Followed by specialized diabetes clinics after emergency department visit |  |  |
| Noor Abdulhadi et al. 2013 | Oman | T2DM | Health providers | Cross sectional study | Quantitative method | Link to care, treatment, medication adherence | The experiences of primary health-care providers of their encounters with patients with type 2 diabetes | Appropriate training for health-care providers in communication skills, decreasing the workload | Workload and lack of teamwork approach, poor patients' management adherence, culture, language barriers, providers' frustration and aggressive attitudes towards the patients |  |
| Korcegez et al. 2017 | Northern Cyprus | T2DM | Health receivers | Interventional study | Quantitative method | Treatment, medication adherence, control | HbA1c, blood pressure, lipid profile, body mass index, waist circumference, medication adherence, self-care activities | Pharmacist-led care program |  |  |
| Ramallo-Fariña et al. 2015 | Spain | T2DM | Health receivers and providers | Interventional study | Mixed methods | Link to care, treatment, medication adherence, control | HbA1c, cardiovascular risk factors, macrovascular and microvascular diabetes complications, quality of life, psychological outcomes, diabetes knowledge, healthcare utilization | Patients are receiving an educational group program and monitored by means of logs and a web-based platform and tailored semi-automated SMS. Primary care professionals are receiving a short educational program, which includes a decision support tool embedded into the electronic clinical record and a monthly feedback report of patients’ results. |  |  |
| Hallberg et al. 2018 | US | T2DM | Health receivers | Interventional study | Quantitative method | Treatment, medication adherence | HbA1c, weight, medication use, fasting serum glucose and insulin, HOMA-IR, blood lipids lipoproteins, liver and kidney function markers, high-sensitivity C-reactive protein, body skeletal muscle mass | A novel care model providing continuous remote care with medication management based on biometric feedback |  |  |
| Andrich et al. 2020 | US | T2DM | Health receivers | Implementation study | Quantitative method | Treatment, medication adherence | HBA1C, fasting blood glucose, quality of life | Information sharing between patients and providers, psychosocial support, behavioral support with lifestyle modification, multi-disciplinary integration, and care coordination |  |  |
| Brain et al. 2019 | US | T2DM | Health receivers | Implementation study | Quantitative method | Treatment, medication adherence | HbA1c, percent of patients who participated in the phone call with provider, comparison of kept, cancelled, and no-show visits of patients enrolled in this project during year of project compared with their visit history the year before, patient and provider satisfaction | Scheduled phone calls, improving patient’s diabetes self-management techniques |  |  |
| Chen et al. 2016 | China | T2DM | Health receivers | Interventional study | Quantitative method | Treatment, medication adherence | The number of essential examinations/tests the patients received, COC, health care outcomes | Pay-for-performance programs |  |  |
| Collier et al. 2014 | US | T2DM | Health receivers | Cohort | Quantitative method | Treatment, medication adherence, control | HbA1c | Integrated a pharmacist into multidisciplinary patient-aligned care teams |  |  |
| Prenissl et al. 2019 | India | T2DM | Health receivers | Cross sectional study | Quantitative method | Awareness, treatment, control | Prevelence of diabetes |  | Living in a rural area, male, income, lower education |  |
| Lee et al. 2018 | Malaysia | T2DM | Health receivers | Cross sectional study | Quantitative method | Treatment, medication adherence, control | The experiences and views of individuals with type 2 diabetes mellitus on their diabetes self-management and potential roles for community pharmacists in diabetes self-management education and support. |  | Misconceptions about diabetes management, a lack of knowledge, not confident about treatment regimens |  |
| Dalal et al. 2014 | US | T2DM | Health receivers | Cohort | Quantitative method | Awareness | Glycemic levels, health care costs |  | Counseling and education |  |
| Hong et al. 2014 | Korea | T2DM | Health receivers | Cohort | Quantitative method | Link to care, medication adherence | Continuity of Care Index, medication possession ratio |  |  | Institution-level continuity of ambulatory care |
| Santos et al. 2019 | Portugal | T2DM | Health receivers | Cross sectional study | Quantitative method | Control | Body mass index, blood pressure, HbA1c, lipid profile | Transition to a different type 2 diabetes physician |  |  |
| Hsu et al. 2016 | China | T2DM | Health receivers | Cohort | Quantitative method | Link to care | High-quality continuity of care |  |  | Younger patients, the oldest (>65 years) group, least severe, with a high number of comorbidities |
| Lee et al. 2012 | Korea | T2DM | Health receivers | Cohort | Quantitative method | Awareness, screen, link to care, control | Prevalence of undiagnosed diabetes | Diabetes information on Internet, holding events on Diabetes Day and through mass-media coverage of high- risk factors of diabetes, preventative diabetes care through diet and health behavior via home visits, early diabetes detection through medical check-ups, standardized self-care program |  |  |
| Joseph et al. 2022 | India | Both | Health providers | Implementation study | Quantitative method | Link to care, treatment | Informational continuity of care | Patient-held health records | High workload, influence of patient behaviors and interruptions in outpatient settings | Beliefs about capabilities, intentions, goals and optimism |
| Geldsetzer et al. 2022 | India | Both | Health receivers | Cross sectional study | Quantitative method | Awareness, diagnosis, link to care, treatment, medication adherence, control | BP, capillary blood glucose |  |  | Female, living in an urban area, increasing household wealth, higher educational attainment |
| Jorgensen et al. 2020 | Zanzibar | Both | Health receivers | Cross sectional study | Quantitative method | Screen, diagnosis, treatment, control | BP, FBG |  | Living in urban area | Female, older age, overweight or obese, former smoker, having a known diagnosis of diabetes |
| Patel et al. 2020 | India | Both | Health receivers | Implementation study | Quantitative method | Screen, diagnosis, treatment, control | Reach, effectiveness, adoption, and implementation of the program components | Integrated Tracking, Referral, and Electronic Decision Support, and Care Coordination (I-TREC) program |  |  |
| Wollum et al. 2018 | South Africa | Both | Health receivers and providers | Cross sectional study | Mixed methods | Diagnosis, treatment, control | Risk factor and disease prevalence, gaps in diagnosis, treatment, and management of these conditions from both the patient and provider perspective | The HealthRise community-based interventions | Long waiting times, lack of transportation, concerns about confidentiality, perceived discrimination, insufficient consultation time with providers, limited human resource capacity and availability of diagnostic equipment at lower-level facilities |  |
| Gabert et al. 2017 | India | Both | Health receivers | Implementation study | Mixed methods | Screen, diagnosis, treatment, control | Gaps in the continuum of care for hypertension and diabetes in two Indian communities | Improved health education and outreach services, screening for those without symptoms | male, lack of diagnostic equipment and testing capabilities, high pricing, Stock-outs of drugs, insufficient time to explain medications or provide counselling, transportation availability and cost | With an elevated blood glucose |
| Odland et al. 2020 | Sierra Leone | Both | Health receivers | Cross sectional study | Quantitative method | Screen, diagnosis, link to care, treatment, control | Prevelence of risk factors, diabetes and hypertension |  | Live in an urban, female | Male, lack of knowledge and cost |
| Khetan et al. 2017 | India | Both | Health receivers | Cross sectional study | Quantitative method | Diagnosis, treatment, control | Prevelence of risk factors, diabetes and hypertension |  | Physician inertia, lack of patient follow-up, pill burden |  |
| Price et al. 2018 | Malawi | Both | Health receivers | Cross sectional study | Quantitative method | Screen, diagnosis, treatment, control | Prevelence of diabetes and hypertension |  | Rural men | Urban women |
| Gee et al. 2012 | Canada | Both | Health receivers | Cross sectional study | Quantitative method | Awareness, treatment, control | Prevelence of diabetes and hypertension |  |  |  |
| Byun et al. 2021 | Korea | Both | Health providers | Cross sectional study | Quantitative method | Awareness, link to care, treatment | Perception of two alternative health care priorities | Health education programs, a community-based intervention program |  | Health education, working continuity of staff, patient satisfaction, healthy lifestyle |
| Osetinsky et al. 2022 | Tanzania | Both | Health receivers | Cross sectional study | Quantitative method | Screen, diagnosis, link to care, treatment, control | Prevelence of diabetes and hypertension |  | Male | Increasing age, completing primary school, with healthcare fee exemptions, people with hypertension who had a prior diagnosis of diabetes, the NHIF enrollment |
| LaMonica et al. 2022 | Samoa | Both | Health receivers | Cross sectional study | Quantitative method | Awareness, diagnosis, treatment, control | Prevelence of diabetes and hypertension |  |  | Female |
| Leniz et al. 2019 | Chile | Both | Health receivers | Cross sectional study | Quantitative method | Diagnosis, link to care, | Proportion of hypertensive and diabetic patients, self-report of diagnosis and treatment, recent foot and ophthalmological exams | Continuity of care | Education, single people, physical activity | Age greater than 65 years, female gender, widowed, retired, having high cardiovascular risk, BMI >30 |
| Jayanna et al. 2019 | India | Both | Health receivers | Cross sectional study | Mixed methods | Link to care, treatment, medication adherence, control | Prevelence of diabetes and hypertension |  | Misconceptions about the causes of illness, anxiety and stigma about the disease, non-acceptance of disease status, fears about medications and side effects, cultural beliefs, social norms and challenges faced with health systems |  |
| Zhao et al. 2022 | China | Both | Health receivers | Cohort | Quantitative method | Awareness, control | Prevelence of hypertension, diabetes, and dyslipidemia |  |  | Males, married, education, high consumption, wealth |
| Dey et al. 2022 | India | Both | Health receivers | Cross sectional study | Mixed methods | Control | HbA1c, blood pressure, risk factors, perspectives of patients and the counsellors about the barriers and enablers for better control |  | Older age, longer duration of disease, additional chronic conditions, tobacco consumption, higher body mass index(BMI), alcohol consumption, lower social groups, stress as a result of family or financial worries, poor lifestyle, and poor health-seeking behavior interplay |  |
| Ramli et al. 2016 | Malaysia | Both | Health receivers | Interventional study | Quantitative method | Control | HbA1c, BP, serum lipid profile, body mass index and waist circumference, medication adherence levels, process of care, prescribing patterns | EMPOWER-PAR: organization of healthcare, self-management support, decision support, delivery system design, community resources and policies |  |  |
